# New insights into the genetics of mandibular retrognathism: novel candidate genes

**DOI:** 10.1007/s00056-023-00512-z

**Published:** 2024-01-31

**Authors:** Eva Paddenberg-Schubert, Erika Küchler, Caio Luiz Bitencourt Reis, Alice Corrêa Silva-Sousa, Christian Kirschneck

**Affiliations:** 1 Department of Orthodontics, University of Regensburg, Franz-Josef-Strauss-Allee 11, 93053 Regensburg, Germany; 2 Department of Orthodontics, University Hospital Bonn, Medical Faculty, Bonn, Germany; 3 Department of Pediatric Dentistry, School of Dentistry of Ribeirão Preto, University of São Paulo, Ribeirão Preto, SP Brazil; 4 Restorative Dentistry Department, School of Dentistry of Ribeirão Preto, University of São Paulo, Ribeirão Preto, SP Brazil

**Keywords:** Orthodontic diagnostics, Skeletal class II malocclusion, Mandible, Single nucleotide polymorphism, Biomarkers, Kieferorthopädische Diagnostik, Skelettale Klasse II, Mandibula, Einzelnukleotid-Polymorphismen, Biomarker

## Abstract

**Purpose:**

Mandibular retrognathism (MR) is a common skeletal malocclusion in humans with a strong genetic component. Single nucleotide polymorphisms (SNPs) in genes encoding epidermal growth factor (EGF) and EGF receptor (EGFR) could be involved in the etiology of mandibular retrognathism. Therefore, in this study, we investigated whether SNPs in the genes encoding for *EGF* and *EGFR* are associated with MR in German teenagers.

**Methods:**

This nested case–control study evaluated German orthodontic patients, aged 10–18 years. DNA, which was isolated from buccal epithelial cells using two cytobrushes, was used for genotyping analysis and digital pretreatment lateral cephalograms were examined to calculate SNB and ANB. Patients with a retrognathic mandible (SNB < 78°) were included as cases, while patients with an orthognathic mandible (SNB = 78–82°) were included as controls. Four SNPs in the genes encoding for *EGF* and *EGFR* were chosen and genotyped using real-time PCR. Allele, genotype, and haplotype frequency were compared across groups (α = 5%).

**Results:**

Finally, 119 patients were included in this study (45 orthognathic mandible, 74 retrognathic mandible). The minor allele G in rs4444903 (*EGF*) was statistically more frequent in individuals with an orthognathic mandible (*p* = 0.008). The haplotype formed by the mutant alleles for rs4444903|rs2237051 (*EGF*; G|A) was statistically more frequent in the orthognathic mandible group (*p* = 0.007). The SNPs rs4444903 and rs2237051 in *EGF*, and rs2227983 in *EGFR* were statistically associated with a decreasing risk of developing a retrognathic mandible according to univariate and multivariate statistical analysis (*p* < 0.05).

**Conclusion:**

SNPs in *EGF* (rs4444903 and rs2237051) and *EGFR* (rs2227983) were associated with MR in our German sample and could be genetic biomarkers for early and individualized diagnostic identification of retrognathic mandibular development by means of genetic screening tests.

**Supplementary Information:**

The online version of this article (10.1007/s00056-023-00512-z) contains supplementary material, which is available to authorized users.

## Abstract (de)

**Hintergrund:**

Die mandibuläre Retrognathie (MR) ist eine beim Menschen häufig auftretende Dysgnathie, welche eine ausgeprägte genetische Komponente aufweist. Einzel-Nukleotid-Polymorphismen (SNPs) in Genen, welche für den epidermalen Wachstumsfaktor (EGF) oder dessen Rezeptor (EFGR) kodieren, könnten ätiologische Faktoren für die MR sein. Das Ziel dieser Studie bestand daher darin, zu untersuchen, ob solche SNPs zur MR in deutschen Jugendlichen eine Assoziation aufweisen.

**Methodik:**

In dieser Fall-Kontroll-Studie wurden kieferorthopädische Patienten im Alter von 10–18 Jahren analysiert. Während zur Analyse der Genotypen DNA aus Speichelproben extrahiert wurde, wurden zur Bestimmung des SNB- und des ANB-Winkels digitale prätherapeutische Fernröntgenseitenbilder (FRS) ausgewertet. Die Patienten, welche einen retrognathen Unterkiefer (SNB < 78°) zeigten, wurden der Fallgruppe zugeschrieben, während solche mit einem orthognathen Unterkiefer (SNB = 78–82°) als Kontrollen eingeschlossen wurden. Vier SNPs für EGF und EGFR wurden ausgewählt und mittels Realtime-PCR genotypisiert. Die Häufigkeit der Allele, der Genotypen und der Haplotypen wurden zwischen den beiden Gruppen verglichen (α-Fehler 5%).

**Ergebnisse:**

119 Patienten, von denen 45 einen orthognathen und 74 einen retrognathen Unterkiefer aufwiesen, wurden final in die Studie eingeschlossen. Bei Patienten mit einem orthognathen Unterkiefer wurden das Minor-Allel G im SNP rs4444903 (EGF) (*p* = 0,008) sowie der durch die mutierten Allele für rs4444903|rs2237051 (EGF; G|A) gebildete Haplotyp (*p* = 0,007) statistisch signifikant häufiger aufgefunden. Gemäß der uni- und multivariaten Analyse waren die SNPs rs4444903 und rs2237051 im *EGF*-Gen und rs2227983 im *EGFR*-Gen statistisch signifikant mit einem reduzierten Risiko für einen retrognathen Unterkiefer assoziiert.

**Schlussfolgerung:**

SNPs im *EGF*- (rs4444903 und rs2237051) und im *EGFR*-Gen (rs2227983) waren in der untersuchten deutschen Population mit einer MR assoziiert und könnten sich daher als genetische Biomarker eignen, um während einer frühen und individualisierten Diagnostik im Rahmen von genetischen Screeningtests einen retrognathen Unterkiefer zu identifizieren.

## Introduction

Mandibular retrognathism is defined as an abnormally posterior positioned mandible in relation to the anterior skull base [3]. Although the relation of the jaw bases and the craniofacial morphology determine an individual’s malocclusion [7], this craniofacial dysgnathia is often associated with a skeletal class II malocclusion, which occurs in about 23–29% of the population worldwide [8]. Mandibular retrognathism has a polygenic etiological background [1, 2, 14, 26]. Across different populations, some genes have been identified as etiological factors of mandibular retrognathism [9].

Single nucleotide polymorphisms (SNPs) are variations in the DNA sequence that occur, when a single nucleotide varies between members of a biological species or paired chromosomes in an individual. SNPs can influence the expression and/or functions of genes and have been explored in complex traits, including skeletal malocclusions and other dentofacial traits [13]. Previous investigations from different research groups revealed that a variety of SNPs are involved in the mandibular retrognathism phenotype [1, 2, 12, 14, 15, 26]. A recent study in a German sample showed an association between a SNP in the gene encoding the transforming growth factor beta receptor type 2 (TGFBR2) with mandibular retrognathism [12].

Growth factors are mostly proteins or steroid hormones that act as signaling molecules regulating many cellular functions such as cell proliferation, survival, and differentiation. Some growth factors stimulate a cellular response by binding to specific receptors [10]. The epidermal growth factor receptor (EGFR) is a receptor tyrosine kinase that is activated by binding of its ligand, the epidermal growth factor (EGF), resulting in receptor dimerization and autophosphorylation, and activation of signaling pathways promoting proliferation. EGF and EGFR play important roles in skeletal biology [22] and their function is necessary for normal craniofacial development [19]. Resorption, formation, and maintenance of bone are coordinated by the action of several hormones, transcription factors, and growth factors [22]. Since growth factors promote the events of cell growth, the investigation of their potential role as predictive biomarkers for skeletal malocclusions is an exciting approach, which could enable early and individualized diagnostic identification of retrognathic mandibular development by means of genetic screening tests in the future. Therefore, in this study, we investigated whether SNPs in the genes encoding *EGF* and *EGFR *are involved in the etiology of mandibular retrognathism of German teenagers.

## Materials and methods

This study was approved by the Human Ethics Committee at the University of Regensburg (number 19-1549-101) and conducted according to the ethical principles of the Helsinki Declaration. Informed consent was obtained from all patients and their parents or legal guardians. Furthermore, an age-appropriate assent document was also used for patients younger than 14 years.

The Strengthening the Reporting of Genetic Association study (STREGA) statement checklist [17] was used to design and report this study, and the checklist is presented in supplementary table 1.

Sample size calculation, recruitment and collection of this nested case–control study were previously described by Kirschneck et al. [12]. Briefly, German orthodontic patients were consecutively recruited during orthodontic treatment in 2020 and 2021, and the sample size was determined with a power of 0.80%, α of 0.05, and an effect size of 0.225.

Adults and patients with syndromes, congenital alterations including dental agenesis of permanent tooth/teeth (except for third molar agenesis), patients with cleft lip and/or palate (syndromic or isolated forms of cleft), and patients with facial trauma were excluded. Furthermore, after the cephalometric analysis, patients with mandibular prognathism (SNB > 82°) were also excluded. Only one individual per family was included to avoid genetic bias. In addition, to minimize genetic and phenotypic variance and maximize data interpretation, only patients with Middle European ancestry were included [12].

All patients included were teenagers not biologically related and age ranged from 10–18 years.

### Cephalometric analysis

Digital pretreatment lateral cephalograms as part of patients’ orthodontic records with the mandible in maximal intercuspation were used in the cephalometric analysis. Measurements were performed by two trained and calibrated orthodontists who presented good interexaminer and intraexaminer reliability as previously reported in Kirschneck et al. [12].

**Fig. 1:**
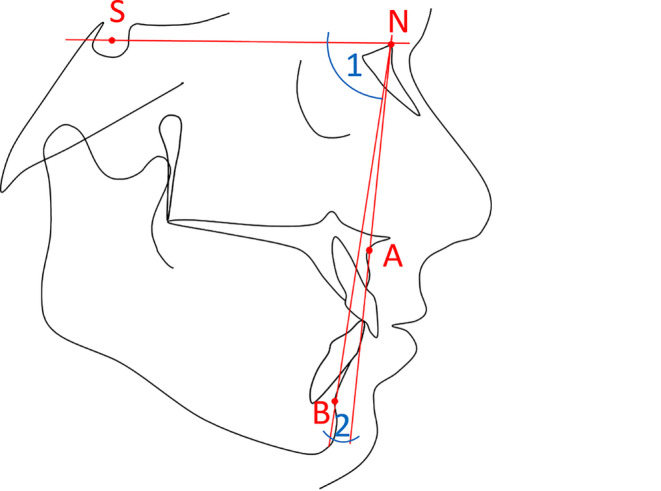
Determination of mandibular retrognathism and skeletal class using cephalometric variables. *S* sella, *N* nasion, *A* point A, *B* point B, *angle 1* SNB (degree of prognathism of the mandible), *angle 2* ANB (skeletal class) Bestimmung einer mandibulären Retrognathie und der skelettalen Klasse anhand kephalometrischer Parameter. *S* Sella, *N* Nasion, *A* A-Punkt, *B* B-Punkt, *Winkel 1* SNB (Prognathiegrad des Unterkiefers), *Winkel 2* ANB (skelettale Klasse)

The radiographs were imported as lossless TIF files into the software ivoris® analyze pro (Computer konkret AG, Falkenstein, Germany, version 8.2.15.110) and calibrated. Cephalometry based on Segner and Hasund [23] was conducted digitally, although only skeletal parameters were considered for analyses. The anatomical landmarks point A, point B, sella (S), and nasion (N) were determined manually using the cephalometric analysis software (ivoris analyze pro), and the angular measurements SNB and ANB were calculated (Fig. 1).

The phenotype definition was as follows: patients with a retrognathic mandible were selected as cases (SNB < 78°), while patients with an orthognathic mandible were selected as controls (SNB = 78–82°). Patients with mandibular prognathism were excluded (SNB > 82°).

### Genetic analysis

We selected candidate SNPs at the *EGF* and *EGFR* genes (Table 1) mostly based on the minor allele frequency reported in European populations (> 20%), the SNPs function, and based on previous results of studies investigating their association with several phenotypes suggesting clinical relevance of these SNPs (http://www.ncbi.nlm.nih.gov/snp/). SNPs in the promoter, coding, and intronic region were selected. The characteristics and description of the SNPs investigated in this study are presented in Table 1.

**Table 1:** Characteristics of studied SNPs Eigenschaften der untersuchten SNPs

Gene	SNPs	Base change	Comment	Function	Genotyping success rate	HWE *p*-value
*EGF*	rs4444903	A > G	Promoter region	EGF levels	0.975	0.074
	rs2237051	G > A	Missense variant	EGF levels	0.950	> 0.999
*EGFR*	rs2227983	G > A	Missense variant	Decreased EGF affinity	0.966	0.824
	rs763317	A > G	Intronic variant	Unknown	0.983	0.709

The meaning and impairment of the SNPs were obtained through the National Center for Biotechnolgy Information (NCBI) and LitVar

*HWE* Hardy–Weinberg equilibrium, *EGF* epidermal growth factor, *EGFR* epidermal growth factor receptor

For the genotyping analysis, genomic DNA was isolated from buccal epithelial cells collected using two cytobrushes placed in extraction solution (Tris-HCl 10 mmol/L, pH 7.8; EDTA 5 mmol/L; SDS 0.5%, 1 mL). Briefly, proteinase K (100 ng/mL) was added to each tube. Ammonium acetate was also added to remove nondigested proteins and the solution was then centrifuged. DNA was precipitated with isopropanol and washed with ethanol. The DNA was quantified by spectrophotometry (Nanodrop 1000; Thermo Scientific, Wilmington, DE, USA) [12].

The selected SNPs were blindly genotyped via real-time polymerase chain reaction (PCR) using the Mastercycler® ep realplex‑S thermocycler (Eppendorf AG, Hamburg, Germany). The TaqMan technology was used. A negative control template was included in each reaction plate. In addition, 10% of the samples were randomly selected for repeated analysis and showed 100% concordance. Patients with not enough DNA or DNA samples that failed to be genotyped were excluded from further analyses.

## Statistical analysis

The success genotyping rate was calculated for each SNP, and the Hardy–Weinberg equilibrium was obtained by Pearson χ^2^ test without correction, which was also used to evaluate the distribution of gender between groups. The Mann–Whitney test compared age and SNB medians.

Allele and haplotype frequency comparisons were performed by PLINK version 1.06 (https://zzz.bwh.harvard.edu/plink/ld.shtml). PLINK compares the frequencies between the major allele by Pearson χ^2^ test without correction and between the expected number of haplotypes by Fisher’s exact test.

The univariate Pearson χ^2^ without correction or Fisher’s exact test were performed for univariate genotypic analysis. For the multivariate analysis of the genotypes between the orthognathic and retrognathic mandible group a Poisson regression, which was adjusted by age, was used. Furthermore, the prevalence ratio (PR) and the 95% confidence interval (CI) were calculated. Statistical Package for Social Sciences (SPSS) version 25.0 (IBM Corp., Armonk, NY, USA) was employed for these analyses. Bilateral *p*-values were adopted for all tests, and *p* < 0.05 indicated a statistically significant difference.

## Results

A total of 119 patients were included in this study (57 males and 62 females). Forty-five had an orthognathic mandible, while 74 showed a retrognathic mandible. Table 2 illustrates the characteristics of the sample. The SNB angle was statistically different between the orthognathic mandible and retrognathic mandible groups (*p* < 0.001).

**Table 2:** Characteristics of the studied sample Eigenschaften des untersuchten Kollektivs

		Total	Orthognathic mandible	Retrognathic mandible	*p*-value
*N* (%)		119 (100)	45 (37.8)	74 (62.2)	–
Gender	Male (%)	57 (47.9)	25 (55.5)	32 (42.2)	0.192
	Female (%)	62 (52.1)	20 (44.5)	42 (57.8)	
Median age (95% CI)		12.31 (12.0–12.68)	12.68 (12.2–13.97)	12.20 (11.3–12.55)	0.034*
Median SNB (95% CI)		76.8 (75.9–77.8)	79.60 (79.10–80.20)	75.20 (74.3–75.9)	<0.001*

Gender was compared between groups by χ^2^ test. Age and SNB were compared between groups by Mann–Whitney test

*95% CI* 95% confidence interval

*p < 0.05

Table 1 shows the details of the studied SNPs and the Hardy–Weinberg equilibrium values for each SNP in the total sample. All SNPs were within the Hardy–Weinberg equilibrium (*p* > 0.05).

The minor allele G in rs4444903 (*EGF*) was statistically more frequent in the orthognathic mandible group compared to the retrognathic group (*p* = 0.008). The haplotype formed by the mutant alleles for rs4444903|rs2237051 (*EGF*; G|A) was statistically more frequent in the orthognathic mandible group in comparison with the retrognathic mandible group (*p* = 0.007; Table 3).

**Table 3:** Allele and haplotype distribution between groups Verteilung der Allele und Haplotypen zwischen den Gruppen

Chromosome	Gene	SNPs	Allele/Haplotypes	Frequency		*p*-value
				Orthognathic mandible	Retrognathic mandible	
4	*EGF*	rs4444903	G	0.477	0.305	0.008*
		rs2237051	A	0.488	0.376	0.096
		rs4444903|rs2237051	G|A	0.387	0.222	0.007*
			A|A	0.113	0.154	0.382
			G|G	0.089	0.089	0.988
			A|G	0.410	0.534	0.071
7	*EGFR*	rs2227983	A	0.352	0.253	0.109
		rs763317	G	0.511	0.423	0.191
		rs2227983|rs763317	A|G	0.187	0.098	0.053
			G|G	0.336	0.317	0.769
			A|A	0.165	0.155	0.838
			G|A	0.312	0.429	0.075

Frequencies between the major alleles were compared by chi-square test and between the expected number of haplotypes by Fisher exact test

*EGF* epidermal growth factor, *EGFR* epidermal growth factor receptor

*p < 0.05

Table 4 shows the uni- and multivariate comparison of the genotypes between groups. The rs4444903 and rs2237051 (*EGF*), and rs2227983 (*EGFR*) SNPs were statistically associated with a decreasing chance of presenting with a retrognathic mandible. In the codominant model, the heterozygous patients for these SNPs had less chance of exhibiting a retrognathic mandible than the dominant homozygous patients. In the dominant model, heterozygous and recessive homozygous patients had less chance of developing a retrognathic mandible than the dominant homozygous patients (*p* < 0.05; PR < 1.0).

**Table 4:** Univariate and multivariate analysis of genotypes comparison between groups Univariate und bivariate Analyse der Genotypen zum Vergleich zwischen den Gruppen

Gene	SNP	Model	Genotype	Orthognathic mandible		Retruded mandible		*p*-value^u^	*p*-value^m^	PR	95% CI
				*n*	%	*n*	%				
*EGF*	rs4444903	Co-Dominant	AA	8	18.18	33	45.83	Reference			
			AG	30	68.18	34	47.22	0.004*	0.011*	0.69	0.52–0.91
			GG	6	13.64	5	6.94	0.020*	0.216	0.64	0.32–1.28
		Dominant	AA	8	18.18	33	45.83	Reference			
			AG + GG	36	81.82	39	54.17	0.003*	0.008*	0.68	0.52–0.90
		Recessive	AA + AG	38	86.36	67	93.06	Reference			
			GG	6	13.64	5	6.94	0.232	0.533	0.80	0.40–1.59
	rs2237051	Co-Dominant	GG	9	20.45	29	42.03	Reference			
			GA	27	61.36	28	40.58	0.013*	0.006*	0.65	0.47–0.88
			AA	8	18.18	12	17.39	0.194	0.278	0.80	0.53–1.19
		Dominant	GG	9	20.45	29	42.03	Reference			
			GA + AA	35	79.55	40	57.97	0.017*	0.007*	0.69	0.52–0.90
		Recessive	GG + GA	36	81.82	57	82.61	Reference			
			AA	8	18.18	12	17.39	0.914	0.941	1.01	0.68–1.50
*EGFR*	rs2227983	Co-Dominant	GG	16	36.36	41	57.75	Reference			
			GA	25	56.82	24	33.80	0.015*	0.019*	0.67	0.48–0.93
			AA	3	6.82	6	8.45	> 0.999^f^	0.818	0.94	0.58–1.52
		Dominant	GG	16	36.36	41	57.75	Reference			
			GA + AA	28	63.64	30	42.25	0.003^f^*	0.028*	0.71	0.53–0.96
		Recessive	GG + GA	41	93.18	65	91.55	Reference			
			AA	3	6.82	6	8.45	> 0.999^f^	0.650	1.11	0.69–1.79
	rs763317	Co-Dominant	AA	11	24.44	22	30.56	Reference			
			AG	22	48.89	39	54.17	0.791	0.328	0.85	0.62–1.17
			GG	12	26.67	11	15.28	0.158	0.056	0.60	0.36–1.01
		Dominant	AA	11	24.44	22	30.56	Reference			
			AG + GG	34	75.56	50	69.44	0.474	0.130	0.78	0.57–1.07
		Recessive	AA + AG	33	73.33	61	84.72	Reference			
			GG	12	26.67	11	15.28	0.131	0.103	0.67	0.41–1.08

Frequencies between the major alleles were compared by chi-square test and between the expected number of haplotypes by Fisher exact test

*EGF* epidermal growth factor, *EGFR* epidermal growth factor receptor

*p < 0.05

## Discussion

Mandibular retrognathism is a common maxillofacial alteration that can cause occlusal problems leading to class II malocclusion. The treatment of class II skeletal malocclusion due to mandibular retrognathism is one of the most common challenges in orthodontic practice. Mandibular retrognathism is also associated with esthetic problems and in severe cases with obstructive sleep apnea [11]. Therefore, studies investigating mandibular retrognathism are extremely important in the orthodontic literature and the number of research groups investigating the genetic background of this condition has been increasing in the past decade. In this study, some SNPs in the encoding genes *EGF* and *EGFR* were associated with mandibular retrognathism.

Mandibular retrognathism was previously associated with SNPs in *MYO1H* [1], *MATN1* [2], *ADAMTS9* [26], *BMP2* [14], *PTH, VDR, CYP24A1*, and *CYP27B1* [15] in different populations. Recently, a study indicated that *TGFBR2* could be involved in mandibular retrognathism, and this finding was also observed in the sample evaluated in the present study [12]. In another study, four SNPs in transforming growth factor beta 1 were investigated: *TGFB1* (rs1800469 and rs4803455) and *TGBR2* (rs3087465 and rs764522), which are members of the growth factor family that has numerous key roles in the bone tissue controlling physiological processes [21]. The authors found that the SNP rs3087465 in *TGFBR2* was associated with mandibular retrognathism [12]. Thus, we raised the hypothesis that other SNPs in growth factors encoding genes could be involved in the etiology of mandibular retrognathism.

Growth and development of the skeletal system is the main component or driver for postnatal somatic growth. During childhood and adolescence, bone lengthening and acquisition of peak bone mass and its trabecular organization are achieved, involving the production of calcified cartilage and its conversion and modeling into trabecular bone. Mandibular condylar cartilage is known as the center of most growth in the craniofacial complex and is associated with maxillofacial skeletal morphogenesis [20]. Although previous studies demonstrated some important functions of EGF and EGF-like ligands in regulating bone growth and modeling, the expression, roles, and action mechanisms of the EGF family of growth factors and its receptor in bone growth regulation are less explored than for other growth factors [27], especially in craniofacial growth and development.

In our research, the minor allele of the two studied SNPs in *EGF *(rs4444903 and rs2237051), as well as their haplotype (G|A), were associated with a decreasing risk of mandibular retrognathism. An in vitro experiment observed that EGF negatively regulated chondrogenesis through the inhibition of precartilage condensation and also by modulating signaling [28]. A study with an animal model also showed that defects in bone lengthening were observed in EGF transgenic mice [5]. EGF level can be modulated by the functional selected SNP in *EGF *at position 61 (A > G; SNP rs4444903), in which the GG genotype has a higher gene expression than the AA genotype [25]. This could explain why the AA genotype was more frequent in patients with a retrognathic mandible. Similar results were observed in Brazilian patients with dentofacial deformities, in which the SNP rs4444903 was involved in mandibular measurements [4].

The rs2237051 SNP in the coding region of the *EGF* gene is a missense substitution (Met708Ile) and was also associated with mandibular retrognathism in our sample. Although this SNP has never been previously explored in craniofacial growth, it has been explored in dental research in past years. The SNP rs2237051 was associated with generalized aggressive periodontitis [16, 26] and was recently associated with an increased risk of peri-implantitis [6]. In our sample, the GG genotype was more common in patients with a retrognathic mandible than in patients with an orthognathic mandible.

We also found an association between the *EGFR* and mandibular retrognathism. We observed that the SNP rs2227983 was involved in the risk of developing a retrognathic mandible. The SNP rs2227983 is located in the coding region of the gene and is a missense substitution at codon 497 (Arg497Lys) that leads to an attenuation in ligand binding and growth stimulation [18]. EGFR is expressed in chondroblasts of the developing ossification centers [27]. An animal model study showed that in *egfr* null mice the growth plate was significantly increased in the region of hypertrophic chondrocytes [24]. Newborn egfr^−/−^ mice presented facial mediolateral defects including narrow, elongated snouts, and an underdeveloped lower jaw [19].

In our study, the three associated SNPs are classified as potentially functional: SNPs that can result in amino acid changes of the corresponding proteins (the missense SNPs), or the SNPs located in the promoter region of the gene and potentially influencing gene expression and EGF levels, which point them as interesting possible biomarkers. Briefly, our research raises potential future research avenues in orthodontic research, since the functional SNPs rs4444903, rs2237051, and rs2227983 could be biomarkers for mandibular retrognathism and should be explored in other populations.

## Conclusion

Single nucleotide polymorphisms in the encoding genes *EGF* and *EGFR* were associated with mandibular retrognathism in a German sample and could be genetic biomarkers for early and individualized diagnostic identification of retrognathic mandibular development by means of genetic screening tests, which could supplement the cephalometric evaluation in young growing children for individualized orthodontic diagnostics, treatment planning, and prognosis.

## Supplementary Information

Supplementary Table 1

## Data Availability

All pertinent data are available from the corresponding author upon reasonable request.

## References

[1] Arun RM, Lakkakula BVKS, Chitharanjan AB (2016) Role of myosin 1H gene polymorphisms in mandibular retrognathism. Am J Orthod Dentofacial Orthop 149:699–704. 10.1016/j.ajodo.2015.10.028 27131252 10.1016/j.ajodo.2015.10.028

[2] Balkhande PB, Lakkakula BVKS, Chitharanjan AB (2018) Relationship between matrilin‑1 gene polymorphisms and mandibular retrognathism. Am J Orthod Dentofacial Orthop 153:255–261.e1. 10.1016/j.ajodo.2017.06.023 29407503 10.1016/j.ajodo.2017.06.023

[3] Bell WH (1966) Surgical correction of mandibular retrognathism. Am J Orthod 52: 518–528. 10.1016/0002-9416(66)90105-9 10.1016/0002-9416(66)90105-9 5220417

[4] Cavalcante RC, Bergamaschi IP, Sebastiani AM et al (2020) Association between facial measurements and polymorphisms in human epidermal growth factor and transforming growth factor β1. Br J Oral Maxillofac Surg 58:214–219. 10.1016/j.bjoms.2019.11.024 31924381 10.1016/j.bjoms.2019.11.024

[5] Chan SY, Wong RW (2000) Expression of epidermal growth factor in transgenic mice causes growth etardation. J Biol Chem 275:38693–38698. 10.1074/jbc.M004189200 11001946 10.1074/jbc.M004189200

[6] Chang Z, Jiang D, Zhang S et al (2021) Genetic association of the epidermal growth factor gene polymorphisms with peri-implantitis risk in Chinese population. Bioengineered 12:8468–8475. 10.1080/21655979.2021.1983976 34592884 10.1080/21655979.2021.1983976 PMC8806989

[7] Enlow DH, Moyers RE, Hunter WS et al. (1969) A procedure for the analysis of intrinsic facial form and growth. An equivalent-balance concept. Am J Orthod 56: 6–23. 10.1016/0002-9416(69)90254-1 10.1016/0002-9416(69)90254-1 5255278

[8] Ferrazzano GF, Cantile T, Sangianantoni G et al (2019) Oral health status and Unmet Restorative Treatment Needs (UTN) in disadvantaged migrant and not migrant children in. Dent, vol 20. Eur J Paediatr, Italy, pp 10–14 10.23804/ejpd.2019.20.01.02 10.23804/ejpd.2019.20.01.02 30919637

[9] Gershater E, Li C, Ha P et al (2021) Genes and Pathways Associated with Skeletal Sagittal Malocclusions: A Systematic Review. Int J Mol Sci 22:13037. 10.3390/ijms222313037 34884839 10.3390/ijms222313037 PMC8657482

[10] Heldin CH (1995) Dimerization of cell surface receptors in signal transduction. Cell 80: 213–223. 10.1016/0092-8674(95)90404-2 10.1016/0092-8674(95)90404-2 7834741

[11] Ishiguro K, Kobayashi T, Kitamura N et al (2009) Relationship between severity of sleep-disordered breathing and craniofacial morphology in Japanese male patients. Oral Surg Oral Med Oral Pathol Oral Radiol Endod 107:343–349. 10.1016/j.tripleo.2008.08.021 18996034 10.1016/j.tripleo.2008.08.021

[12] Kirschneck M, Zbidat N, Paddenberg E et al (2022) Transforming Growth Factor Beta Receptor 2 (TGFBR2) Promoter Region Polymorphisms May Be Involved in Mandibular Retrognathism. Biomed Res Int 2022:1503052. 10.1155/2022/1503052 35757474 10.1155/2022/1503052 PMC9217526

[13] Küchler EC, Henklein SD, Proff P et al (2022) Single Nucleotide Polymorphisms in COX2 Is Associated with Persistent Primary Tooth and Delayed Permanent Tooth Eruption. Int J Environ Res Public Health 19:10047. 10.3390/ijerph191610047 36011680 10.3390/ijerph191610047 PMC9408601

[14] Küchler EC, Reis CLB, Carelli J et al (2021) Potential interactions among single nucleotide polymorphisms in bone- and cartilage-related genes in skeletal malocclusions. Orthod Craniofac Res 24:277–287. 10.1111/ocr.12433 33068497 10.1111/ocr.12433

[15] Küchler EC, Reis CLB, Marañón-Vásquez G et al (2021) Parathyroid Hormone Gene and Genes Involved in the Maintenance of Vitamin D Levels Association with Mandibular Retrognathism. J Pers Med 11:369. 10.3390/jpm11050369 34063310 10.3390/jpm11050369 PMC8147469

[16] Li W, Wang X, Tian Y et al (2020) A novel multi-locus genetic risk score identifies patients with higher risk of generalized aggressive periodontitis. J Periodontol 91:925–932. 10.1002/JPER.19-0135 31833563 10.1002/JPER.19-0135

[17] Little J, Higgins JPT, Ioannidis JPA et al (2009) STrengthening the REporting of Genetic Association Studies (STREGA)—an extension of the STROBE statement. Genet Epidemiol 33:581–598. 10.1002/gepi.20410 19278015 10.1002/gepi.20410

[18] de Mattia E, Roncato R, Palazzari E et al (2020) Germline and Somatic Pharmacogenomics to Refine Rectal Cancer Patients Selection for Neo-Adjuvant Chemoradiotherapy. Front Pharmacol 11:897. 10.3389/fphar.2020.00897 32625092 10.3389/fphar.2020.00897 PMC7311751

[19] Miettinen PJ, Chin JR, Shum L et al (1999) Epidermal growth factor receptor function is necessary for normal craniofacial development and palate closure. Nat Genet 22:69–73. 10.1038/8773 10319864 10.1038/8773

[20] Mizoguchi I, Toriya N, Nakao Y (2013) Growth of the mandible and biological characteristics of the mandibular condylar cartilage. Jpn Dent Sci Rev 49:139–150. 10.1016/j.jdsr.2013.07.004

[21] Poniatowski ŁA, Wojdasiewicz P, Gasik R et al (2015) Transforming growth factor Beta family: insight into the role of growth factors in regulation of fracture healing biology and potential clinical applications. Mediators Inflamm 2015:137823. 10.1155/2015/137823 25709154 10.1155/2015/137823 PMC4325469

[22] Schneider MR, Sibilia M, Erben RG (2009) The EGFR network in bone biology and pathology. Trends Endocrinol Metab 20:517–524. 10.1016/j.tem.2009.06.008 19819718 10.1016/j.tem.2009.06.008

[23] Segner D, Hasund A (2003) Individualisierte Kephalometrie, 4th edn. Segner, Hamburg

[24] Sibilia M, Wagner B, Hoebertz A et al (2003) Mice humanised for the EGF receptor display hypomorphic phenotypes in skin, bone and heart. Development 130:4515–4525. 10.1242/dev.00664 12925580 10.1242/dev.00664

[25] Tanabe KK, Lemoine A, Finkelstein DM et al (2008) Epidermal growth factor gene functional polymorphism and the risk of hepatocellular carcinoma in patients with cirrhosis. JAMA 299:53–60. 10.1001/jama.2007.65 18167406 10.1001/jama.2007.65

[26] Wang X, Li W, Xu L et al (2020) The association of EGF rs2237051 variant, serum EGF levels and generalized aggressive periodontitis: a preliminary study. PeerJ 8: e9212. 10.7717/peerj.9212 10.7717/peerj.9212 PMC7243814 32477838

[27] Xian CJ (2007) Roles of epidermal growth factor family in the regulation of postnatal somatic growth. Endocr Rev 28:284–296. 10.1210/er.2006-0049 17322455 10.1210/er.2006-0049

[28] Yoon YM, Oh CD, Kim DY et al (2000) Epidermal growth factor negatively regulates chondrogenesis of mesenchymal cells by modulating the protein kinase C‑alpha, Erk‑1, and p38 MAPK signaling pathways. J Biol Chem 275:12353–12359. 10.1074/jbc.275.16.12353 10766877 10.1074/jbc.275.16.12353

